# Association Between Head Computed Tomography Findings and In-Hospital Mortality in COVID-19 Patients

**DOI:** 10.7759/cureus.54339

**Published:** 2024-02-16

**Authors:** Kensaku Yoshida, Mikio Nakajima, Richard H Kaszynski, Masayoshi Horino, Takuma Higo

**Affiliations:** 1 Neurological Surgery, Tokyo Metropolitan Hiroo Hospital, Tokyo, JPN; 2 Clinical Epidemiology and Health Economics, Tokyo University, Tokyo, JPN; 3 Emergency and Critical Care Center, Tokyo Metropolitan Hiroo Hospital, Tokyo, JPN

**Keywords:** low-density lesions, cerebral infarction, basal ganglia, ct, cerebral small vessel disease, covid-19

## Abstract

Objective: The present study investigated the association between head computed tomography (CT) findings and mortality in patients with COVID-19. Specifically, we focused on low-density lesions identified on head CT screenings.

Materials and methods: We performed a single-center, retrospective cohort study based on data obtained from the medical charts of inpatients admitted to the Tokyo Metropolitan Hiroo Hospital between January 1 and December 31, 2021. We focused on the basal ganglia--a representative anatomical region for assessing routine head CT in patients with COVID-19. Patients were divided into two groups based on the presence or absence of low-density lesions in the basal ganglia. The primary outcome was all-cause in-hospital mortality, and the secondary outcome was the length of hospital stay. We performed multivariable regression analyses for outcomes to adjust for patients’ background and disease severity.

Results: During the study period, 1,906 COVID-19 patients were admitted to our facility. Among them, 1,203 patients underwent head CT evaluations and were included in this study. The median age was 56 years (interquartile range: 43-76 years) and 725 patients (60.3%) were male. A total of 235 (19.5%) patients required oxygen therapy on admission and 1,051 (87.4%) patients had pneumonia. Crude in-hospital mortality was 6.1% and the median length of hospital stay was 10 days (interquartile range: 8-14 days). The multivariate regression analyses showed that low-density lesions in the basal ganglia were significantly associated with increased in-hospital mortality and prolonged hospital stay.

Conclusions: The presence of ischemic changes in the basal ganglia denoted by low-density findings may be a promising prognostic factor in patients with COVID-19.

## Introduction

Under the global pandemic era, COVID-19-related thrombosis has emerged as a noteworthy sequela contributing to increased disease severity and mortality [1]. The risk factors known to contribute to severe forms of COVID-19 include older age and the presence of one or more comorbidities (e.g., diabetes mellitus, hypertension, malignancy, cardiovascular disease, cerebrovascular disease, and obesity). A higher number of risk factors has been attributed to a greater risk of developing more severe forms of disease and also carries a proportionally higher mortality [2-5].

Among the several known risk factors contributing to severe COVID-19, cerebrovascular disease is documented as an independent risk factor; however, it remains challenging to elucidate the presence of cerebrovascular disease in patients with no documented history [6]. Middle-aged and older adults are often asymptomatic and imaging studies only coincidentally reveal discernable radiographic changes indicative of ischemic change in the brain [7,8]. One of the hallmarks of ischemic change as observed via head computed tomography (CT) is the presence of low-density lesions in the basal ganglia (BG).

To our knowledge, the association between head CT findings and clinical outcomes in COVID-19 patients has not been thoroughly investigated at the fine- or large-scale levels. Therefore, the present study aimed to elucidate the association between the presence of low-density lesions in the BG and clinical outcomes in hospitalized COVID-19 patients.

## Materials and methods

Setting and patients

We performed a single-center, retrospective cohort study based on data obtained from the medical charts of inpatients admitted to the Tokyo Metropolitan Hiroo Hospital between January 1 and December 31, 2021. Hiroo Hospital is one of the designated tertiary emergency medical centers in Tokyo and has primarily been tasked with the management of COVID-19.

We included all patients with COVID-19 admitted to our hospital during the defined study period. Patients who did not receive head and chest CT were excluded from the study. Patients with missing data required for analyses were also excluded. All COVID-19 patient diagnoses were confirmed by a reverse transcription polymerase chain reaction test for SARS-CoV-2 via a nose or throat swab, sputum, or saliva sample.

Variables

Patient characteristics, medical history, oxygen requirement on admission, laboratory data on admission, and outcome data were obtained from electronic hospital medical records. Patient characteristics included age, sex, and body mass index. Medical histories included in the present study were hypertension, diabetes mellitus, cardiovascular disease, chronic kidney disease, chronic obstructive pulmonary disease, and admission from a nursing facility. Laboratory data on the day of admission included the following: D-dimer, serum creatinine, C-reactive protein, and glycosylated hemoglobin. These variables have been reported as risk factors for death due to COVID-19 [9].

Computed tomography

**Figure 1:**
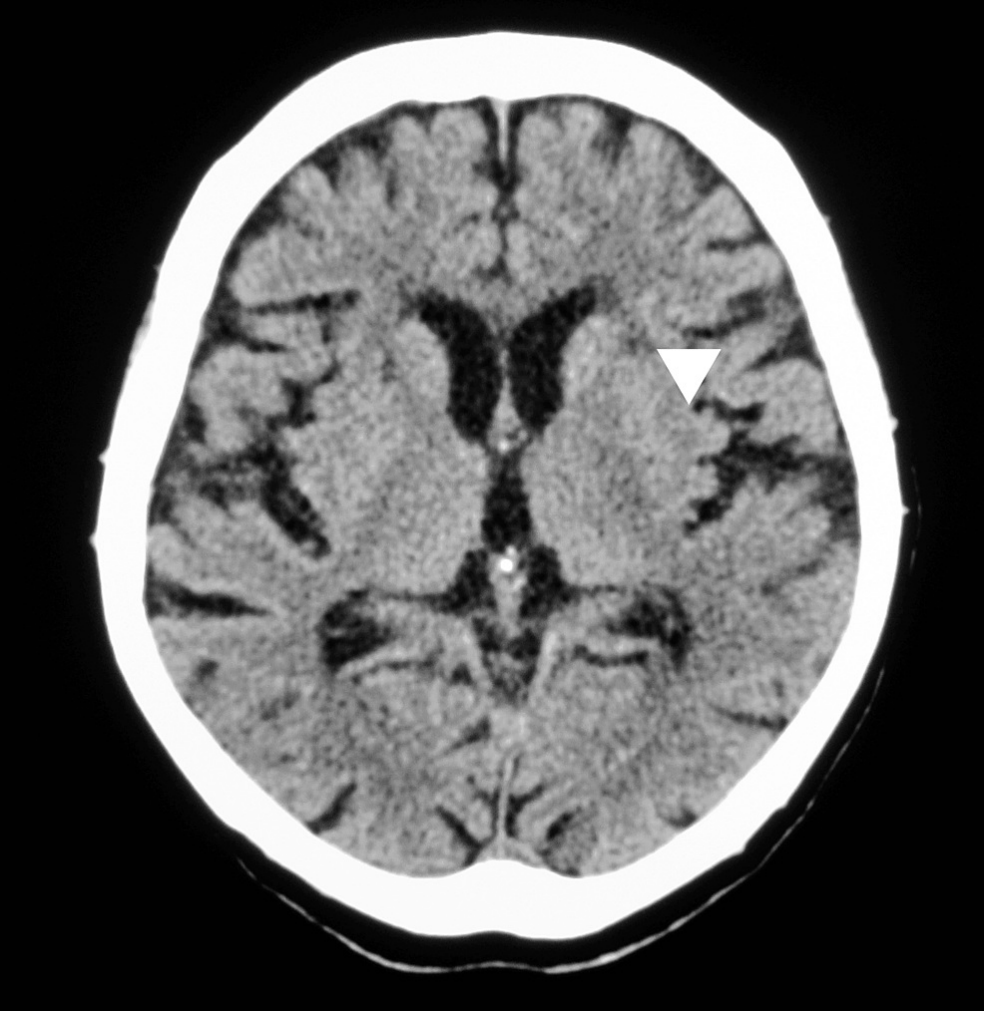
Head CT The white triangle indicated the low-density lesion at the left basal ganglia on head CT.

Chest CT was routinely performed to detect pneumonia in our facilities in patients with COVID-19 on admission, except for patients who were pregnant or refused CT examination. Additionally, head CT was concurrently performed to rule out the possibility of intracranial hemorrhage in the vast majority of COVID-19 patients. Chest CT findings were reviewed for the presence of pneumonia by the radiology department. Head CT was performed using a standard protocol. Head CT findings included (i) low-density lesions in the BG (Figure 1), (ii) low-density change in the deep white matter, (iii) periventricular lucency, (iv) old infarction, and (v) acute intracranial bleeding. Head CT findings were evaluated by a neurosurgeon board certified by the Japan Neurosurgical Society.

Exposure and outcomes

The presence of low-density lesions in the BG was considered the exposure in the present study. One or more low-density lesions in the BG were interpreted as “present” and none as “absent.” The Fazekas and age-related white matter changes (ARWMC) scales were used for this determination [10]. The primary outcome was all-cause in-hospital mortality and the secondary outcome was length of hospital stay. We compared these outcomes between patients with low-density lesions in the BG (low-BG group) and those without (control group).

Statistical analysis

Continuous variables are presented as medians with interquartile ranges (IQRs), and categorical variables are reported as counts and percentages. We compared patient backgrounds between the low-BG group and the control group. The chi-squared test or Fisher's exact test was used to compare proportions. Wilcoxon rank-sum test was adopted to compare continuous variables. To examine the association between CT findings of low-density lesions in the BG and in-hospital mortality, a multivariable logistic regression analysis was conducted. Similarly, a multivariable linear regression analysis was conducted for the length of hospital stay as a dependent variable. Independent variables included in these regression models were age per 10 years, sex, admission from a nursing facility, hypertension, diabetes mellitus, oxygen requirement on admission, D-dimer, and C-reactive protein. The value of D-dimer was divided into ≥1 mg/dL or <1 mg/dL [11]. The value of C-reactive protein was divided into ≥2.69 mg/dL or <2.69 mg/dL [12]. Odds ratios and their 95% confidence intervals (CIs) were calculated for mortality and absolute differences, and their 95% CIs were calculated for length of hospital stay. Multicollinearity between the independent variables was checked using the variance inflation factor (VIF) < 2.5 [13].

As a sensitivity analysis, we calculated E-values to assess the robustness of the results to potential residual or unmeasured confounders [14]. The E-value is the minimum strength of association that unmeasured confounders would need to have with both the exposure and the outcome to explain away a treatment-outcome association. E-values focus on the magnitude of the confounder associations that could produce confounding bias equal to the observed treatment-outcome association. A large E-value implies that considerable unmeasured confounding would be needed to explain away an effect estimate. A p-value of < 0.05 was considered statistically significant. All analyses were performed using Stata MP15 (StataCorp, College Station, TX) and R version 4.1.2 (The R Foundation, Vienna, Austria).

Ethics

This study was conducted in compliance with the Strengthening the Reporting of Observational Studies in Epidemiology (STROBE) statement and was approved by the Institutional Review Board of Tokyo Metropolitan Hiroo Hospital (approval number: J-4). Owing to the anonymous nature of the retrospective data, we obtained informed consent in the form of an opt-out on the hospital's website.

## Results

**Figure 2:**
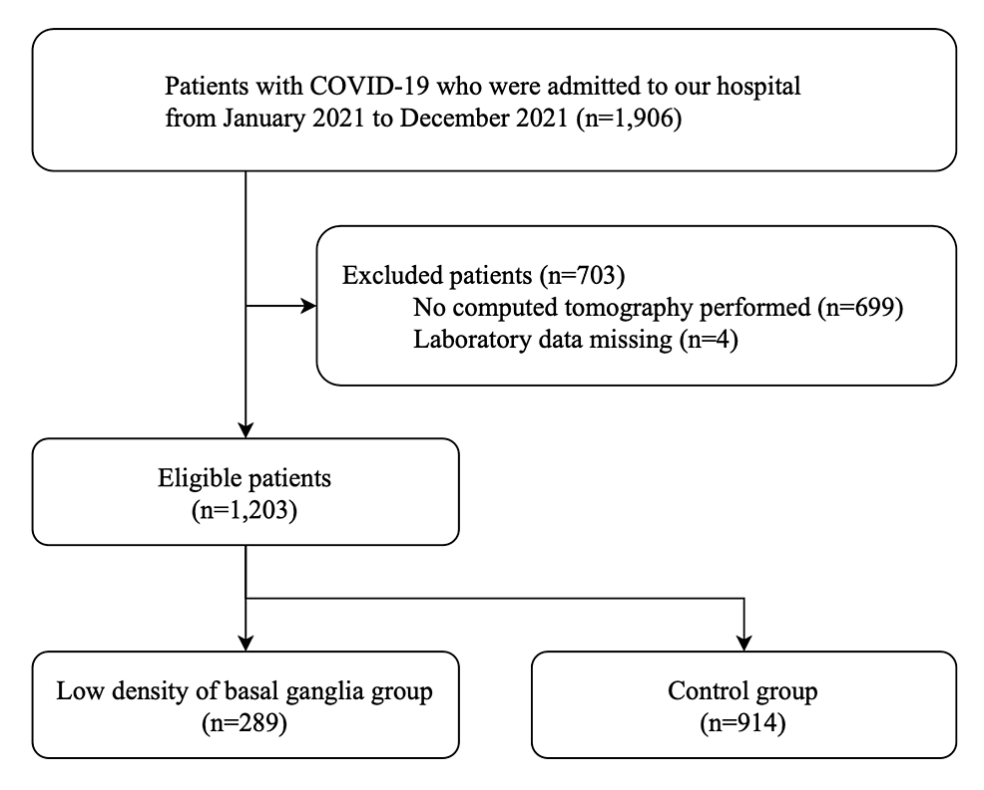
Study flowchart

During the study period, 1,906 consecutive patients were admitted to our facility for COVID-19. After application of the inclusion and exclusion criteria, we extracted data from 1,203 patients (Figure 2). Clinical background and laboratory findings are reported in Table 1. The median age was 56 years (IQR, 43-76 years) and 725 patients (60.3%) were male. A total of 235 (19.5%) patients required oxygen therapy on admission and 1,051 (87.4%) patients had pneumonia. Crude in-hospital mortality was 6.1% (73/1,203). The median length of hospital stay was 10 days (IQR, 8-14 days). The low-BG group was significantly older, presented with more complications, and had a higher ratio of severe patients in comparison with the control group. In-hospital mortality was 3.2% (29/914) in the control group and 15.2% (44/289) in the low-BG group. The median length of hospital stay was 10 days (IQR, 7-12 days) in the control group and 13 days (IQR, 10-20 days) in the low-BG group.

**Table 1:** Patient characteristics * No low-density lesions of basal ganglia in head CT. Data are shown as numbers (%) unless otherwise specified.

Variables	Total (n = 1,203)		Control* (n = 914)		Low-density lesions in basal ganglia (n = 289)		P-value
Age, years, median (IQR)	56	(43-76)	52	(40-63)	80	(70-88)	<0.001
Male	725	(60.3)	580	(63.5)	145	(50.2)	<0.001
Past medical history							
Hypertension	329	(27.3)	194	(21.2)	135	(46.7)	<0.001
Diabetes mellitus	303	(25.2)	206	(22.5)	97	(33.6)	<0.001
Cardiovascular disease	86	(7.1)	38	(4.2)	48	(16.6)	<0.001
Chronic kidney disease	31	(2.6)	16	(1.8)	15	(5.2)	0.001
Chronic obstructive pulmonary disease	47	(3.9)	38	(4.2)	9	(3.1)	0.42
Body mass index category							
<18.5	115	(9.6)	64	(7.0)	51	(17.6)	<0.001
18.5–24.9	633	(52.6)	471	(51.5)	162	(56.1)	
25.0–29.9	309	(25.7)	255	(27.9)	54	(18.7)	
≥30.0	132	(11.0)	117	(12.8)	15	(5.2)	
Missing	14	(1.2)	7	(0.8)	7	(2.4)	
Admission from a nursing facility	121	(10.1)	50	(5.5)	71	(24.6)	<0.001
Oxygen requirement on admission	235	(19.5)	161	(17.6)	74	(25.6)	0.003
Laboratory findings on admission, median (IQR)							
D-dimer, mmol/L	0.9	(0.7-1.3)	0.9	(0.7-1.2)	1.2	(0.8-2.1)	<0.001
Serum creatinine, mg/dL	0.83	(0.68-1.01)	0.82	(0.68-0.98)	0.88	(0.71-1.13)	<0.001
C-reactive protein, mg/dL	3.69	(1.04-7.83)	3.70	(1.04-7.65)	3.66	(1.05-8.45)	0.40
Glycosylated hemoglobin, %	6.0	(5.7-6.4)	6.0	(5.7-6.3)	6.0	(5.7-6.5)	0.030
Pneumoniae in chest CT on admission	1,051	(87.4)	802	(87.7)	249	(86.2)	0.48
Abnormal findings on head CT							
Low-density lesions in deep white matter	334	(27.8)	137	(15.0)	197	(68.2)	<0.001
Periventricular lucency	332	(27.6)	126	(13.8)	206	(71.3)	<0.001
Old infarction	76	(6.3)	15	(1.6)	61	(21.1)	<0.001
Intracranial bleeding	5	(0.4)	1	(0.1)	4	(1.4)	0.003

Table 2 demonstrates the result of a multivariable logistic regression analysis for in-hospital mortality. The low-BG group was significantly associated with higher in-hospital mortality (odds ratio, 1.84; 95% confidence interval, 1.03-3.27; p = 0.040). The result of a multivariable linear regression analysis for the length of hospital stay is shown in Table 3. The low-BG group was significantly associated with longer length of hospital stay (coefficient, 2.59; 95% confidence interval, 1.46-3.72; p < 0.001). The VIFs were <2.5 for all of the independent variables.

**Table 2:** Results of multivariable logistic regression analysis for in-hospital mortality

Variables	Odds ratio	95% CI	P-value
Low-density lesions in basal ganglia	1.88	1.05–3.37	0.035
Age/10 years	1.81	1.45–2.26	<0.001
Male	1.55	0.87–2.76	0.14
Hypertension	1.20	0.70–2.07	0.51
Diabetes mellitus	1.31	0.75–2.28	0.35
Cardiovascular disease	1.02	0.48–2.14	0.97
Chronic obstructive pulmonary disease	1.71	0.56–5.17	0.34
Body mass index ≥30	1.40	0.49–4.03	0.53
Oxygen requirement on admission	4.80	2.73–8.45	<0.001
D-dimer ≥1 mg/dL	1.35	0.71–2.57	0.36
C-reactive protein ≥2.69 mg/dL	1.83	0.91–3.71	0.09

**Table 3:** Results of multivariable regression analysis for the length of hospital stay

Variables	Coefficient	95% CI	P-value
Low-density lesions in basal ganglia	2.50	1.37 to 3.64	<0.001
Age,/10 years	1.21	0.93 to 1.50	<0.001
Male	0.65	-0.24 to 1.54	0.15
Hypertension	-0.28	-1.30 to 0.74	0.59
Diabetes mellitus	1.38	0.36 to 2.40	0.008
Cardiovascular disease	1.18	-0.49 to 2.85	0.17
Chronic obstructive pulmonary disease	-0.91	-3.06 to 1.24	0.41
Body mass index ≥30	0.79	-0.60 to 2.18	0.27
Oxygen requirement on admission	0.54	-0.56 to 1.65	0.34
D-dimer ≥1 mg/dL	-0.51	-1.45 to 0.42	0.28
C-reactive protein ≥2.69mg/dL	0.79	-0.16 to 1.75	0.10

In the logistic regression model, the E-value was 2.08, and the lower limit of the 95% CI closest to the null point was 1.11. In the linear regression model, the E-value was 2.00, and the lower limit of the 95% CI closest to the null point was 1.62.

## Discussion

This is the first study to establish an association between radiographic head evaluations and clinical outcomes in patients with COVID-19. The presence of ischemic changes in the BG detected incidentally on head CT was independently associated with increased in-hospital mortality and length of hospital stay after adjusting for other risk factors.

Prior studies have shown that older age is the most important risk factor for severe COVID-19 [15]. Data from Wuhan, China reported that among critically ill patients treated intensively with COVID-19, 25% had heart disease and 16.7% had cerebrovascular disease [16]. There are also reports that a history of cerebrovascular disease was associated with unfavorable outcomes in COVID-19 patients and that primary and secondary prevention strategies targeting cardiovascular disease risk factors may improve the outcome of COVID-19 [6,17]. On the other hand, there are no studies establishing asymptomatic cerebral ischemia as a risk factor for severe COVID-19 or mortality. Asymptomatic cerebral ischemic lesions may be incidentally discovered during unrelated or routine radiographic screening efforts.

We considered a number of possibilities that may potentially explain the association between the head CT findings and COVID-19 outcomes. First, hypertension is widely known as a risk factor for small vessel disease; however, hypertension presents with few apparent symptoms. Therefore, the present patient population may include cases with undiagnosed hypertension. Hypertension is reported to exacerbate the severity of COVID-19 disease, mainly through inflammation and inducing excessive immune response. The combination of undiagnosed hypertension with COVID-19 could result in increased damage to multiple systems and organs [18]. COVID-19 is known to have adverse effects on the vascular endothelium and has been shown to cause vascular disorders such as cerebral and myocardial infarctions [19]. Therefore, the underlying presence of vascular damage as small vessel disease appearing as low-density lesions may be a tell-tale sign indicative of severe COVID-19 risk. Second, cytokine storm-induced prothrombotic and coagulation dysfunction triggered by COVID-19 is known to cause microvascular and circulatory disorders [20]. The presence of small vessel disease leads to vascular fragility, which can be severely compromised by COVID-19-associated microcirculatory disturbances.

Even in patients presenting with no apparent legacy risk factors for severe COVID-19, neurosurgeons should be mindful of low-density BG findings and consider these radiographic indicators as potential risk factors for severe COVID-19 disease. Given the results presented in this study, aggressive preventive and therapeutic strategies are encouraged in patients presenting with low-density BG lesions.

There are several limitations that should be noted in the present study. First, this study is a single-center observational study conducted in Japan. Although we included over 1,200 patients, the generalizability and applicability of the results to other facilities and countries might be limited. It should, however, be noted that Japan has the most CT scanners per unit population in the world, which enabled this unique study to be conducted. Second, there might be unmeasured confounders between exposure and outcomes due to the retrospective nature of the study. To resolve this issue, we apply the use of multivariable regression models. Using these models, we adjusted numerous variables that may have already been known as risk and prognostic factors for COVID-19. In addition, we used the E-value to robust our study results described above. The third limitation involves the difficulty of quantitatively assessing low-density lesions via head CT. As a result, future analysis will be based on qualitative head CT studies.

## Conclusions

The presence of ischemic changes in the BG, detected incidentally on head CT, was associated with increased in-hospital mortality and length of hospital stay. There have been no previous reports on the use of head CT images related to the severity of COVID-19. It could be a useful tool for determining prognosis.
